# Two new species of *Simulium* (*Gomphostilbia*) (Diptera, Simuliidae) from Peninsular Malaysia, with keys to Peninsular Malaysian members of the *Simulium ceylonicum* species-group

**DOI:** 10.3897/zookeys.118.1552

**Published:** 2011-07-13

**Authors:** H. Takaoka, M. Sofian-Azirun, R. Hashim, Z. Ya’cob

**Affiliations:** 1 Institute of Biological Sciences, Faculty of Science, University of Malaya, Kuala Lumpur, 50603, Malaysia; 2 Department of Infectious Disease Control, Faculty of Medicine, Oita University, Hasama, Yufu City, Oita, 879-5593 Japan

**Keywords:** black fly, *Gomphostilbia*, Simuliidae, Malaysia

## Abstract

Two new species of black flies, *Simulium (Gomphostilbia) roslihashimi* **sp. n.** and *Simulium (Gomphostilbia) lurauense* **sp. n.**, are described on the basis of reared adult, pupal and larval specimens collected from Peninsular Malaysia. These two new species are placed in the *ceylonicum* species-group within the subgenus *Gomphostilbia*. *Simulium (Gomphostilbia) roslihashimi* **sp. n.** is most distinctive with the male having almost entirely yellow antennae, and *Simulium (Gomphostilbia) lurauense* **sp. n.** is characterized in the female by having the elongate sensory vesicle and the yellowish-white hairs on the base of the costal vein and on the stem vein, in the male by the greater number of large upper-eye facets and the spindle-shaped hind basitarsi which are much narrower than the hind tibiae and femora and in the pupa by the small terminal hooks. Keys to species of the *ceylonicum* species-group reported from Peninsular Malaysia are provided for females, males, pupae and mature larvae.

## Introduction

The fauna of black flies (Diptera: Simuliidae) in Peninsular Malaysia is represented by 37 named and 3 unnamed species, which are all classified in the genus *Simulium* Latreille s. l. and are further placed in four subgenera: 1 species in *Daviesellum* Takaoka & Adler, 18 species (17 named and 1 unnamed) in *Gomphostilbia* Enderlein, 4 species in *Nevermannia* Enderlein, and 17 species (15 named and 2 unnamed) in *Simulium* Latreille s. str. (Crosskey 1973; Edwards 1928; Takaoka 2000, 2008; Takaoka and Adler 1997; Takaoka and Davies 1995, 1997; Takaoka et al. 2010, 2011).

In recent surveys on pupae and larvae of black flies in Peninsular Malaysia, we collected two new species, both of which are assignable to the subgenus *Gomphostilbia*, redefined by Takaoka (2003), by having a bare pleural membrane and a haired katepisternum in the female and male adults, grapnel-shaped hooklets on each side of the ninth pupal abdominal segment and a hypostoma with smooth lateral margins in the larva, and further, within the subgenus, are placed in the *ceylonicum* species-group by having an enlarged male hind basitarsus, a diagnostic morphological characteristics separating this species-group from other species-groups (Takaoka 2003).

These two new species are very similar in the arrangement of the eight pupal gill filaments, one of the key morphological features most frequently used to identify the species in the pupal stage, but are readily distinguishable in the adult stage from each other. The pupal gills of these two new species bear medium-long common basal stalks (i.e., longer than 3/5 of the interspiracular trunk but shorter than the latter), as do those of *Simulium (Gomphostilbia) asakoae* Takaoka & Davies, 1995, one of the five previously described Peninsular Malaysian species of the *ceylonicum* species-group. However, the medium-long common basal stalk can separate these two new species from the remaining four known species, i.e., *Simulium (Gomphostilbia) sheilae* Takaoka & Davies, 1995, which has a short common basal stalk (i.e., shorter than 3/5 of the interspiracular trunk), *Simulium (Gomphostilbia) hoiseni* Takaoka, 2008, *Simulium (Gomphostilbia) longitruncum* Takaoka & Davies, 1995 and *Simulium (Gomphostilbia) sofiani* Takaoka & Hashim, 2011, each of which bears a long common basal stalk (i.e., as long as or longer than the interspiracular trunk) (Takaoka and Davies 1995; Takaoka 2008; Takaoka et al. 2011).

These two new species are described here on the basis of female and male adults reared from pupae, pupal exuviae with their cocoons preserved in 80% ethanol and mature larvae preserved in acetic alcohol solution (1 part of acetic acid: 3 parts of 95% ethanol).

Keys to members of the *ceylonicum* species-group reported from Peninsular Malaysia are provided for females, males, pupae and mature larvae.

The methods of collections, descriptions and illustrations as well as terms for morphological features used here follow those of Takaoka (2003).

The holotype and paratypes will be deposited at the Institute of Biological Sciences, Faculty of Science, University of Malaya, Kuala Lumpur, Malaysia.

## Systematics

### Simulium (Gomphostilbia) roslihashimi

Takaoka & Sofian-Azirun sp. n.

urn:lsid:zoobank.org:act:04DA1BEC-7446-423A-9FFB-43EE4263BB57

http://species-id.net/wiki/Simulium_(Gomphostilbia)_roslihashimi

#### Description.

**Figure 1.**
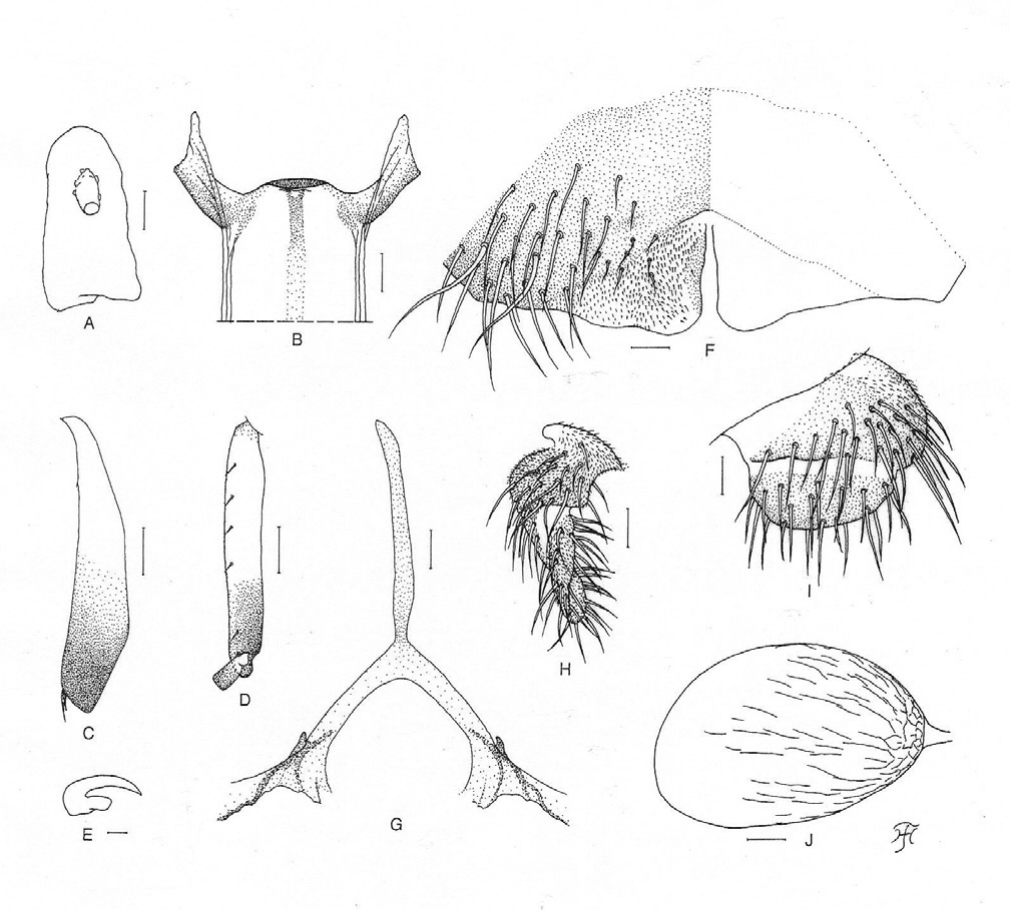
Female of *Simulium (Gomphostilbia) roslihashimi* sp. n. **A** 3rd segment of right maxillary palp with sensory vesicle (front view) **B** cibarium (front view) **C** left hind tibia (outer view) **D** basitarsus and 2nd tarsomere of left hind leg showing calcipala and pedisulcus (outer view) **E** claw **F** sternite 8 and ovipositor valves (ventral view) **G** genital fork (ventral view) **H, I** right paraprocts and cerci (**H** ventral view **I** lateral view) **J** spermatheca (lateral view). Scale bars. 0.1 mm for C and D; 0.02 mm for A, B and F–J; 0.01 mm for E.

 **Female**. Body length 2.2 mm. Head. Slightly narrower than width of thorax. Frons black, slightly shiny when illuminated at certain angle of light, densely covered with whitish-yellow scale-like recumbent short hairs interspersed with few dark simple longer hairs along each lateral margin; frontal ratio 1.64:1.00:2.16; frons-head ratio 1.00:4.31. Fronto-ocular area well developed, narrow, directed dorsolaterally. Clypeus black, slightly shiny when illuminated at certain angle of light, densely covered with yellow hairs interspersed with several dark longer hairs on each side. Labrum 0.55 times as long as clypeus. Antenna composed of scape, pedicel and 9 flagellomeres, dark brown except scape, pedicel, and basal 1/2 of 1st flagellomere yellow. Maxillary palp composed of 5 segments, light to medium brown, proportional lengths of 3rd, 4th, and 5th segments 1.00:1.20:2.62; 3rd segment (Fig. 1A) somewhat swollen; sensory vesicle (Fig. 1A) medium-sized (0.26 times as long as 3rd segment), with medium-sized opening. Maxillary lacinia with 12 inner and 13 outer teeth. Mandible with 22 or 23 inner teeth and lacking outer teeth though outer margin with several very low round ridges at some distance from apex. Cibarium (Fig. 1B) medially forming sclerotized plate folded forward from posterior margin, with moderately sclerotized medial longitudinal ridge. Thorax**.** Scutum dark brown except anterolateral calli dark ochreous, with 5 brownish-black longitudinal vittae (1 narrow median, 2 slightly wider submedian and 2 widest lateral), median and submedian vittae united posteriorly to brownish-black prescutellar area, and submedian and lateral vittae united anteriorly to each other near anterior calli on each side; scutum shiny when illuminated at certain angle of light, densely covered with yellow scale-like recumbent hairs except whitish hairs near anterior and lateral margins. Scutellum medium brown, shiny when illuminated at certain angle of light, covered with yellow short hairs and dark brown long upright hairs along posterior margin. Postnotum dark brown, shiny when illuminated at certain angle of light and bare. Pleural membrane bare. Katepisternum medium to dark brown, longer than deep, shiny when illuminated at certain angle of light, moderately covered with fine short hairs. Legs. Foreleg: coxa yellow; trochanter yellow except apical portion somewhat darkened; femur light brown (though somewhat darkened toward apex, and extreme apex yellowish); tibia white except apical 1/4 brownish-black, with whitish sheen on outer surface of basal 3/4; tarsus black, with moderate dorsal hair crest; basitarsus moderately dilated, 6.14 times as long as its greatest width. Midleg: coxa medium brown except posterior surface brownish-black; trochanter yellow; femur light to medium brown with base and extreme apical tip yellowish; tibia medium to dark brown with basal 1/3 or little more whitish-yellow, covered with whitish fine hairs on basal 2/3 and white sheen on posterior surface of basal 2/3 when illuminated at certain angle of light; tarsus brownish-black except basal 1/2 of basitarsus dark yellow. Hind leg: coxa light brown; trochanter yellow; femur medium brown with base yellow and apical cap dark brown (though extreme apical tip yellowish); tibia (Fig. 1C) light to dark brown with basal 1/2 white, covered with whitish fine hairs on outer and posterior surfaces of basal 3/4 and white sheen on posterior surface of basal 3/4 when illuminated at certain angle of light; tarsus brownish-black except basal 2/3 of basitarsus (though base light brown) and little less than basal 1/2 of 2nd tarsomere white; basitarsus (Fig. 1D) narrow, nearly parallel-sided, 5.80 times as long as wide, and 0.70 and 0.53 times as wide as greatest width of tibia and femur, respectively; calcipala (Fig. 1D) slightly shorter than width at base, and 0.53 times as wide as greatest width of basitarsus. Pedisulcus (Fig. 1D) well defined. Claw (Fig. 1E) with large basal tooth 0.50 times as long as claw. Wing. Length 1.9 mm. Costa with dark spinules and hairs except hairs on basal portion yellow. Subcosta with dark hairs except near apex bare. Hair tuft on stem vein whitish-yellow. Basal portion of radius fully haired; R1 with dark spinules and hairs; R2 with hairs only. Basal cell absent. Haltere. White except basal stem darkened. Abdomen. Basal scale ochreous, with fringe of whitish-yellow hairs. Dorsal surface of abdomen dark brown to brownish-black except basal 1/2 of segment 2 yellow, moderately covered with yellow fine short hairs and dark short to long hairs; tergites of segments 2 and 6–9 shiny when illuminated at certain angle of light. Ventral surface of segments 2 and 3 entirely whitish-yellow, and those of other segments light to dark brown; sternal plate on segment 7 undeveloped. Genitalia. Sternite 8 (Fig. 1F) bare medially, with 25 or 26 medium-long to very long hairs together with few slender short hairs on each side. Ovipositor valves (Fig. 1F) tongue-like, thin, membranous, moderately covered with microsetae interspersed with 2 or 3 short hairs; inner margins slightly concave, somewhat sclerotized, and moderately separated from each other. Genital fork (Fig. 1G) of usual inverted-Y form, with slender stem; arms of moderate width, moderately folded medially; lateral plate of each arm with thin lobe directed medioposteriorly and small stout projection directed anterodorsally. Paraproct in ventral view (Fig. 1H) concave anterolaterally, with 3 or 4 sensilla on anteromedial surface; paraproct in lateral view (Fig. 1I) somewhat produced ventrally, 0.61 times as long as wide, with 24–26 medium-long to long hairs on ventral and lateral surfaces. Cercus in lateral view (Fig. 1I) short, rounded posteriorly, 0.38 times as long as wide. Spermatheca (Fig. 1J) ellipsoidal, 1.55 times as long as its greatest width, well sclerotized except duct and small area near juncture with duct unsclerotized, and with many fissures on surface; internal setae absent; both accessory ducts slender, subequal in diameter to major one.

**Figure 2.**
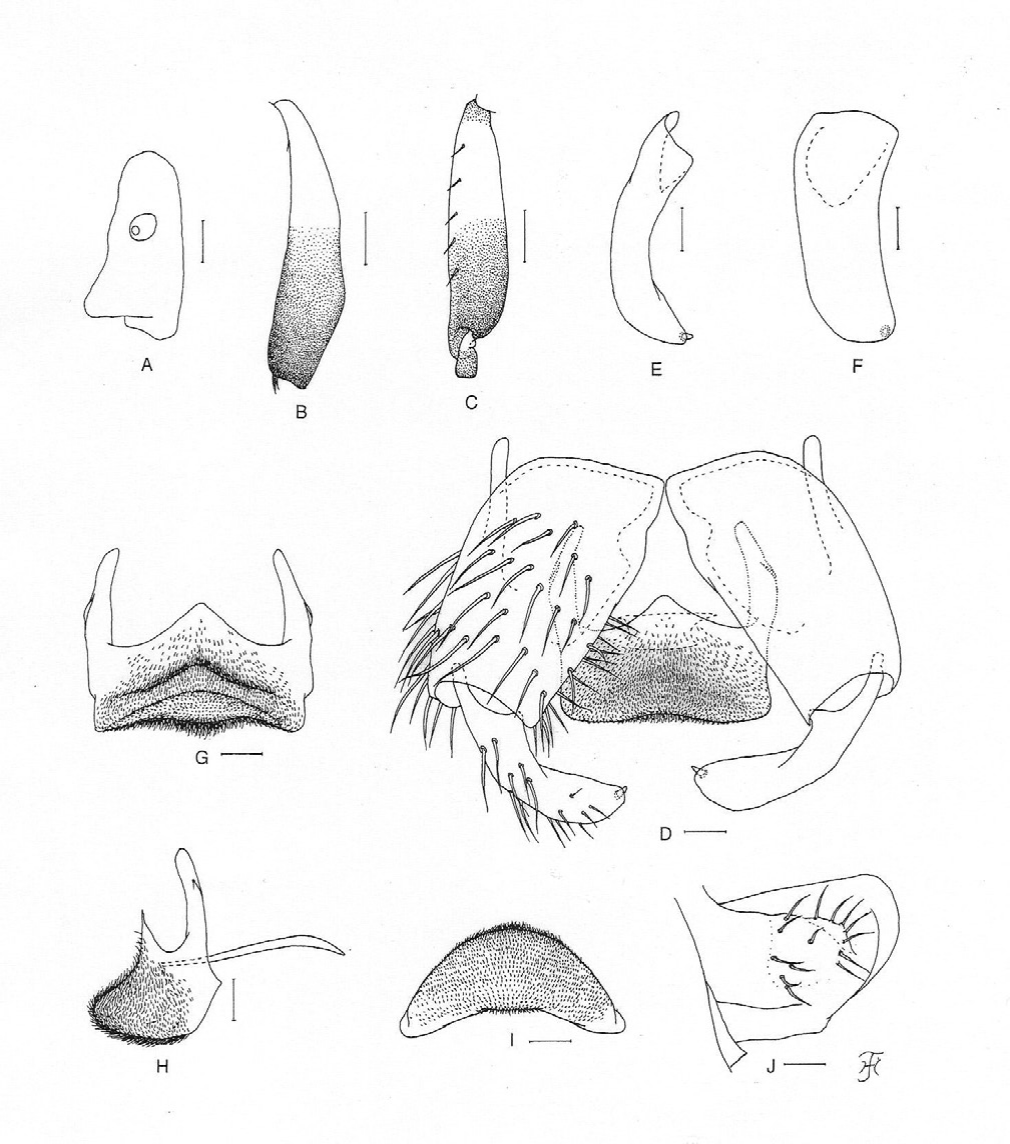
Male of *Simulium (Gomphostilbia) roslihashimi* sp. n.**A** 3rd segment of left maxillary palp with sensory vesicle (front view) **B** left hind tibia (outer view) **C** basitarsus and 2nd tarsomere of left hind leg showing calcipala and pedisulcus (outer view) **D** coxites, styles, ventral plate and median sclerite (ventral view) **E, F** right styles (**E** medial view **F** ventrolateral view) **G** ventral plate (ventral view, though posterior portion slightly inclined ventrally) **H** ventral plate and median sclerite (lateral view) **I** ventral plate (end view) **J** 10th abdominal segment and cercus (right side and lateral view). Scale bars. 0.1 mm for B and C; 0.02 mm for A and D–J.

**Male**. Body length 2.5 mm. Head. Somewhat wider than thorax. Upper eye dark brown, consisting of 11 or 12 vertical columns and 12 (rarely 13) horizontal rows of large facets. Face black, grayish-white pruinose. Clypeus black, whitish pruinose, densely covered with golden-yellow scale-like medium-long hairs (mostly directed upward) interspersed with several dark brown simple longer hairs. Antenna composed of scape, pedicel and 9 flagellomeres, pale yellow to yellow though few apical flagellomeres sometimes slightly to somewhat grayish; 1st flagellomere elongate, 1.78 times as long as 2nd one. Maxillary palp light to medium brown, with 5 segments, proportional lengths of 3rd, 4th, and 5th segments 1.00:1.14:2.95; 3rd segment (Fig. 2A) widened apically; sensory vesicle (Fig. 2A) globular, small (0.15 times as long as 3rd segment), and with very small opening. Thorax. Scutum nearly as in female except median vitta of 5 dark longitudinal vittae often indistinct and short hairs on scutum golden yellow. Legs. Foreleg: coxa yellow; trochanter yellow with some portions light brown; femur light brown though apical tip yellowish; tibia white with subbasal portion on posterior surface grayish and apical 1/4 brownish-black, and covered with yellow hairs on basal 3/4 and white sheen on basal 3/4 when illuminated at certain angle of light; tarsus brownish-black; basitarsus moderately dilated, 6.62– 6.83 times as long as its greatest width. Midleg: coxa medium brown except posterior surface brownish-black; trochanter yellow; femur light brown with base and extreme apical tip yellow; tibia medium brown to brownish-black except basal 1/3 yellow; tarsus dark brown to brownish-black except anterior surface of little less than basal 1/2 of basitarsus dark yellow to light brown. Hind leg: coxa dark yellow to light brown; trochanter yellow; femur light brown with base yellow and apical cap dark brown (though extreme apical tip yellow); tibia (Fig. 2B) dark brown to brownish-black except basal 1/2 (or little less) yellow; tarsus (Fig. 2C) medium to dark brown except basal 1/2 (or little less) of basitarsus whitish-yellow and little less than basal 1/2 of 2nd tarsomere white; basitarsus (Fig. 2C) enlarged, spindle-shaped, 3.78–4.07 times as long as wide, and 0.88–0.94 and 0.91–0.95 times as wide as greatest width of tibia and femur, respectively; calcipala (Fig. 2C) nearly as long as wide, and 0.35 times as wide as greatest width of basitarsus. Pedisulcus (Fig. 2C) well defined. Wing. Length 1.6–1.7 mm. Costa with dark brown spinules as well as dark brown hairs except basal portion with patch of yellowish hairs. Subcosta with or without hairs: i.e., among 7 males examined, 5 males each with 1–4 hairs on each subcosta, 1 male bare on right subcosta and with 1 hair on left subcosta, and 1 male without hair on either right or left subcosta. Hair tuft on stem vein yellow. Basal portion of radius fully haired; R1 with dark spinules and hairs; R2 with hairs only. Basal cell absent. Haltere. Grayish-white except outer surface ochreous and basal stem darkened. Abdomen. Basal scale dark brown, with fringe of light to medium brown hairs. Dorsal surface of abdomen medium brown to brownish-black except segment 2 yellow (though posterior 1/2 of dorsal surface medium brown), covered with dark brown short to long hairs; segments 2 and 5–8 each with pair of shiny dorsolateral or lateral patches; ventral surface of segment 2 yellow, those of segments 3 and 4 yellow except sternites medium brown, and those of other segments medium to dark brown. Genitalia. Coxite in ventral view (Fig. 2D) nearly rectangular, 1.64 times as long as its greatest width. Style in ventral view (Fig. 2D) bent inward, slightly tapered from base toward middle, then slightly widened, tapered to round apex and with apical spine; style in medial view (Fig. 2E) 0.84 times as long as coxite, gently bent inward, nearly parallel-sided from base to apical 3/4, then tapered to apex, and with apical spine; style in ventrolateral view (Fig. 2F) very slightly tapered toward apical 3/4, with rounded apex. Ventral plate in ventral view (Fig. 2D) with body transverse, 0.59 times as long as wide, nearly parallel-sided, with anterior margin produced anteromedially, and posterior margin slightly concave medially (though posterior margin slightly convex medially when ventral plate is slightly inclined – Fig. 2G), densely covered with microsetae on ventral surface; basal arms of moderate length, directed forward, then slightly convergent apically; ventral plate in lateral view (Fig. 2H) moderately produced ventrally; ventral plate in end view (Fig. 2I) rounded ventrally, densely covered with microsetae on posterior surface. Median sclerite (Fig. 2D,H) thin, plate-like, wide. Paramere of moderate size, with 3 distinct long and stout hooks and several smaller ones. Aedeagal membrane moderately setose, slightly sclerotized at base but dorsal plate not well defined. Ventral surface of abdominal segment 10 without distinct hairs near posterior margin. Cercus in lateral view (Fig. 2J) small, rounded, with 13 or 14 hairs.

**Figure 3.**
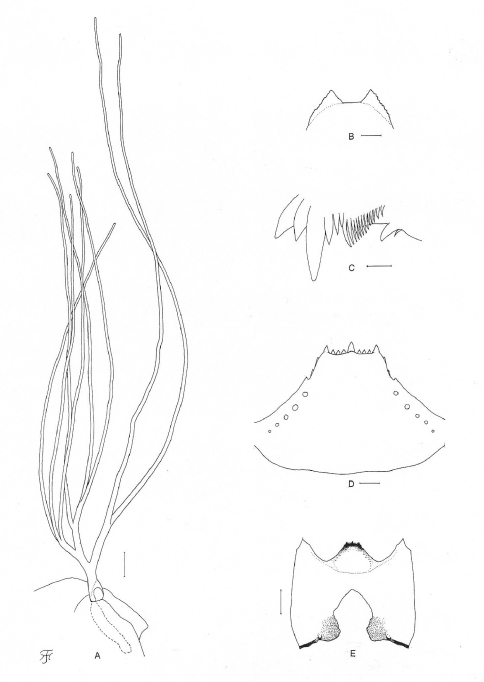
Pupa and larva of *Simulium (Gomphostilbia) roslihashimi* sp. n. **A, B** pupa **C–E** larva **A** right gill filaments (outer view) **B** terminal hooks (end view) **C** left mandible (lateral view) **D** hypostoma (ventral view) **E** head capsule showing postgenal cleft and hypostoma (ventral view). Scale bars. 0.1 mm for A and E; 0.02 mm for B and D; 0.01 mm for C.

**Pupa**. Body length 2.4–2.6 mm. Head. Integument light yellow, moderately covered with small round tubercles; antennal sheath without any protuberances; face with pair of simple very long trichomes with uncoiled apices, and frons with 3 pairs of simple very long trichomes with coiled or uncoiled apices; 3 frontal trichomes on each side arising close together, subequal in length to one another and slightly longer than facial one. Thorax. Integument yellow, moderately covered with round tubercles, with 3 simple very long dorsomedial trichomes with coiled or uncoiled apices, 2 simple very long anterolateral trichomes (1 with coiled apex, 1 with uncoiled apex), 1 simple medium-long mediolateral trichome with uncoiled apex, and 3 simple ventrolateral trichomes with uncoiled apices (1 medium-long and 2 short) on each side. Gill (Fig. 3A) composed of 8 slender thread-like filaments, arranged in [(2+1) (or 3)+(1+2) (or 3)]+2 filaments from dorsal to ventral, with medium-long common basal stalk having somewhat swollen transparent organ ventrally (often partially broken) at base; common basal stalk 0.66–0.72 times as long as interspiracular trunk; dorsal and middle triplets sharing short stalk; dorsal and middle triplets each composed of 1 individual and 2 paired filaments with short primary stalk and very short to short secondary stalk or 3 filaments arising at same level from short to medium-long primary stalk; ventral paired filaments with medium-long stalk which is 1.11–1.47 times as long as common basal stalk and 0.72–1.06 times as long as interspiracular trunk; stalk of ventral pair 1.07–1.33 times as thick as primary stalk of middle triplet, 1.14–1.33 times as thick as primary stalk of dorsal triplet, and 0.89–1.00 times as thick as common stalk of middle and dorsal triplets; primary stalk of dorsal triplet lying against stalk of lower pair at angle of 60–90 degrees when viewed laterally; all filaments grayish light brown, gradually tapered toward apex; 6 filaments of dorsal and middle triplets subequal in length (1.6–2.0 mm long including their own stalks and basal common stalk) and thickness to one another; 2 filaments of ventral pair subequal in length (2.4–3.0 mm long including their own stalk and common basal stalk) and thickness to each other, and 1.50–1.85 times as thick as those of 6 other filaments when compared basally; cuticle of all filaments with well-defined annular ridges and furrows though becoming less marked apically, densely covered with minute tubercles. Abdomen. Dorsally, segments 1 and 2 not pigmented and without tubercles; segment 1 with 1 simple slender medium-long hair-like seta on each side; segment 2 with 1 simple slender medium-long hair-like seta and 5 very short somewhat spinous setae submedially on each side; segments 3 and 4 each with 4 hooked spines and 1 very short somewhat spinous seta on each side; segment 5 lacking spine-combs; segments 6–9 each with spine-combs in transverse row (though those on segment 9 slightly smaller than those on segment 8) (in 1 female pupal exuviae, spine-combs absent on segment 9) and comb-like groups of minute spines on each side; segment 9 with pair of triangular flat terminal hooks of which outer margin is 2.00–3.08 times as long as inner margin and crenulated (Fig. 3B). Ventrally, segment 4 with 1 simple hook and few simple slender very short setae on each side; segment 5 with pair of bifid hooks submedially and few very short simple slender setae on each side; segments 6 and 7 each with pair of bifid inner and simple outer hooks somewhat spaced from each other and few very short simple slender setae on each side; segments 4–8 with comb-like groups of minute spines. Each side of segment 9 with 3 grapnel-shaped hooklets. Cocoon. Wall pocket-shaped, thinly and moderately woven, widely extended ventrolaterally; anterior margin somewhat thickly woven, with dorsal portion not or slightly produced anteriorly when viewed dorsally; posterior 1/2 with floor roughly or moderately woven; individual threads visible; 3.0–3.5 mm long by 2.2–2.5 mm wide.

**Mature larva**. Body length 4.5–4.8 mm. Body creamy to light ochreous except proleg grayish, ventral surface of thoracic segments 2 and 3 grayish or ochreous, and abdominal segments 1–4 almost entirely light grayish; body with reddish-brown markings, i.e., thoracic segment 1 encircled with broad transverse band (though disconnected ventrally), abdominal segments 3 and 4 each with dorsolateral spot on each side (though those on segment 3 very faint or absent), abdominal segments 5 and 6 each with W-shaped broad transverse band on dorsal and dorsolateral surfaces of posterior 1/2 of each segment, abdominal segments 7 and 8 each with broad transverse band almost entirely covering dorsal and dorsolateral surfaces, and abdominal segment 7 with transverse band on ventral surface. Cephalic apotome pale yellow, and moderately covered with simple minute setae; head spots indistinct or very faintly positive. Lateral surface of head capsule pale yellow except eye-spot region whitish, and very sparsely covered with simple minute setae; spots indistinct. Ventral surface of head capsule pale yellow except somewhat darkened area near posterior margin on each side of postgenal cleft, and very sparsely covered with simple minute setae. Antenna composed of 3 segments and apical sensillum, somewhat longer than stem of labral fan; proportional lengths of 1st, 2nd, and 3rd segments 1.00:0.79:0.89. Labral fan with 31 main rays. Mandible (Fig. 3C) with 3 comb-teeth decreasing in length from 1st to 3rd; mandibular serration composed of 2 teeth (1 medium-sized and 1 small); major tooth at acute angle against mandible on apical side; supernumerary serrations absent. Hypostoma (Fig. 3D) with row of 9 apical teeth; median and each corner tooth prominent (though median tooth slightly longer than corner teeth) and much longer than 3 intermediate teeth on each side; lateral margin smooth; 5 hypostomal bristles per side lying parallel to lateral margin. Postgenal cleft (Fig. 3E) lanceolate, 3.1 times as long as postgenal bridge. Cervical sclerite composed of 2 very pale small pieces, not fused to occiput, widely separated medially from each other. Thoracic cuticle bare. Abdominal cuticle almost bare except few posterior segments sparsely to moderately covered with simple minute setae dorsally and dorsolaterally and last segment densely covered with colorless simple setae on each side of anal sclerite. Rectal scales absent. Rectal papilla compound, each of 3 lobes with 5–8 finger-like secondary lobules. Anal sclerite of usual X-form, with anterior arms as long as or little longer than posterior ones, broadly sclerotized at base; accessory sclerite absent. Last abdominal segment expanded ventrolaterally forming double bulges on each side, visible as large conical ventral papilla when viewed from side. Posterior circlet with 96 rows of up to 15 hooklets per row.

#### Type specimens.

 Holotype male (with associated pupal exuviae and cocoon) (preserved in 80% ethanol) reared from pupa, collected from a stream (width 0.5–1.0 m, water temperature 23.0°C, shaded, altitude 390 m) moderately flowing in a forest, Kota Gelanggi, Jerantut, Pahang, Malaysia, 6.III.2011, by M. Sofian-Azirun. Paratypes: 2 female, 6 males with associated pupal exuviae and cocoons, 1 pupal exuviae and cocoon, all preserved in 80% ethanol, and 3 mature larvae preserved in acetic alcohol, same data as those of the holotype.

#### Biological notes.

 The pupae and larvae of this new species were collected from dead tree leaves in the water. Associated species were *Simulium (Gomphostilbia) gombakense*Takaoka & Davies, 1995 and *Simulium (Gomphostilbia) whartoni* Takaoka & Davies, 1995.

**Etymology**. The species name *roslihashimi* is in honor of Prof. Dr. Rosli Hashim, Head of Institute of Biological Sciences, Faculty of Science, University of Malaya, who greatly contributed to the field of ecology of insects and other animals in the tropics.

**Remarks**. This new species is most striking with the male having almost entirely yellowish antennae. None of the known species of the *ceylonicum* species-group (of which the male is known) described from Peninsular Malaysia and other countries (Adler and Crosskey 2011; Takaoka et al. 2011) has such yellowish antennae, although *Simulium (Gomphostilbia) hoiseni* bears yellow to ochreous antennae with the apical 4 or 5 flagellomeres darkened (Takaoka 2008). However, the male of *Simulium (Gomphostilbia) roslihashimi* sp. n. is easily distinguished from *Simulium (Gomphostilbia) hoiseni* by having the hind basitarsus (Fig. 2C) narrower than the hind tibia (Fig. 2B) and the ventral plate parallel-sided when viewed ventrally (Fig. 2D,E) (cf., the hind basitarsus is slightly wider than the hind tibia and the ventral plate is widened posteriorly in *Simulium (Gomphostilbia) hoiseni*).

On the other hand, the female of *Simulium (Gomphostilbia) roslihashimi* sp. n. is very similar to those of *Simulium (Gomphostilbia) asakoae* and *Simulium (Gomphostilbia) longitruncum* in many characteristics including the medium-sized sensory vesicle (Fig. 1A) and the yellow tuft hairs of the stem vein, but is barely distinguished from the latter two species by the outer margin of the mandible without any teeth and the genital fork with a short projection on each arm (Fig. 1G).

This new species is distinguished from other Peninsular Malaysian members of the *ceylonicum* species-group as shown in the keys.

### Simulium (Gomphostilbia) lurauense

Takaoka, Sofian-Azirun & Hashim sp. n.

urn:lsid:zoobank.org:act:4C9E0E9F-1A3C-4186-858A-EF1B42809C87

http://species-id.net/wiki/Simulium_(Gomphostilbia)_lurauense

#### Description.

**Figure 4.**
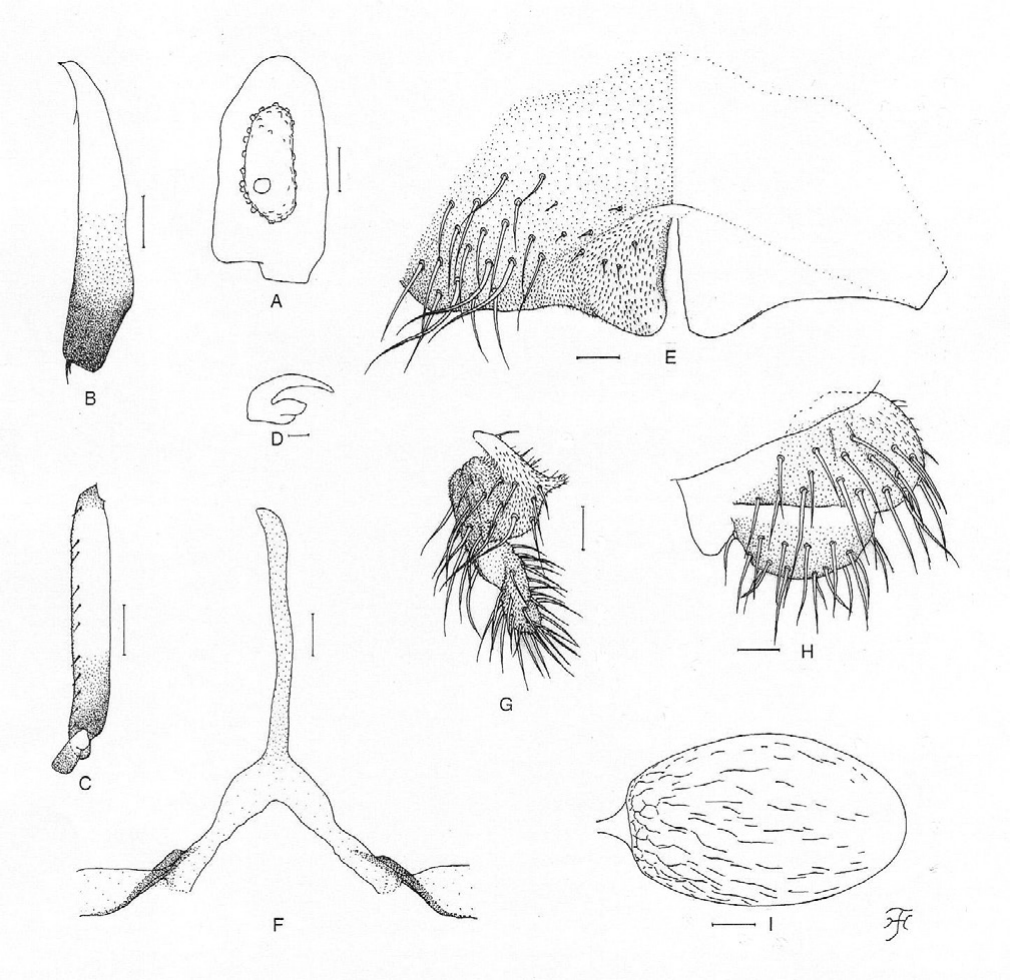
Female of *Simulium (Gomphostilbia) lurauense* sp. n. **A** 3rd segment of right maxillary palp with sensory vesicle (front view) **B** left hind tibia (outer view) **C** basitarsus and 2nd tarsomere of left hind leg showing calcipala and pedisulcus (outer view) **D** claw **E** sternite 8 and ovipositor valves (ventral view) **F** genital fork (ventral view) **G, H** right paraprocts and cerci (**G** ventral view **H** lateral view) **I** spermatheca (lateral view). Scale bars. 0.1 mm for B and C; 0.02 mm for A and E–I; 0.01 mm for D.

 **Female**. Body length 1.9 mm. Head. Slightly narrower than width of thorax. Frons black, very densely covered with whitish-yellow scale-like recumbent short hairs interspersed with 1 dark simple longer hair on upper part near left lateral margin; frontal ratio 1.89:1.00:2.51; frons-head ratio 1.00:4.21. Fronto-ocular area well developed, narrow, directed dorsolaterally. Clypeus black, densely covered with yellow hairs interspersed with several dark longer hairs on each side. Labrum 0.57 times as long as clypeus. Antenna composed of scape, pedicel and 9 flagellomeres, brownish-black except scape, pedicel, and base of 1st flagellomere yellow. Maxillary palp composed of 5 segments, light to medium brown, proportional lengths of 3rd, 4th, and 5th segments 1.00:1.00:2.33; 3rd segment (Fig. 4A) somewhat swollen; sensory vesicle (Fig. 4A) elongate (0.50–0.54 times as long as 3rd segment), with medium-sized opening. Maxillary lacinia with 10 or 12 inner and 14 or 15 outer teeth. Mandible with 28 inner teeth and 6 outer teeth at some distance from apex. Cibarium similar to that of *Simulium (Gomphostilbia) roslihashimi* sp. n. (Fig. 1B). Thorax. Scutum dark brown to brownish-black except anterolateral calli ochreous, shiny when illuminated at certain angle of light, with faintly discernible black longitudinal vittae (1 median and 2 submedian), densely covered with whitish-yellow scale-like recumbent hairs. Scutellum dark brown, shiny when illuminated at certain angle of light, covered with whitish-yellow short hairs and dark brown long upright hairs along posterior margin. Postnotum brownish-black, shiny when illuminated at certain angle of light, and bare. Pleural membrane bare. Katepisternum dark brown, longer than deep, shiny when illuminated at certain angle of light, moderately covered with fine short hairs. Legs. Foreleg: coxa and trochanter whitish-yellow; femur light brown, somewhat darkened toward apex, and with apical cap medium brown, and inner surface of basal portion whitish-yellow; tibia white except apical 1/4 brownish-black, with whitish sheen on outer surface of basal 3/4; tarsus brownish-black to black, with moderate dorsal hair crest; basitarsus moderately dilated, 5.66 times as long as its greatest width. Midleg: coxa dark brown; trochanter yellow; femur light to medium brown except basal 1/4 yellowish; tibia medium to dark brown with basal 2/5 whitish, covered with whitish fine hairs on basal 3/4 and white sheen on posterior surface of basal 3/4 when illuminated at certain angle of light; tarsus dark brown to brownish-black except basal 1/3 of basitarsus yellow. Hind leg: coxa medium brown; trochanter yellow; femur medium brown with base yellow and apical cap dark brown (though extreme tip yellowish); tibia (Fig. 4B) light to dark brown with basal 1/2 white to yellowish-white, covered with whitish fine hairs on basal 3/4 and white sheen on posterior surface of basal 3/4 when illuminated at certain angle of light; tarsus dark brown except basal 2/3 of basitarsus (though base medium brown) and basal 1/2 of 2nd tarsomere yellowish-white; basitarsus (Fig. 4C) narrow, nearly parallel-sided, 6.33 times as long as wide, and 0.64 and 0.53 times as wide as greatest width of tibia and femur, respectively; calcipala (Fig. 4C) slightly shorter than width at base, and 0.56 times as wide as greatest width of basitarsus. Pedisulcus (Fig. 4C) well defined. Claw (Fig. 4D) with large basal tooth 0.56 times as long as claw. Wing. Length 1.9 mm. Costa with dark spinules and hairs except patch of hairs on basal portion yellowish-white. Subcosta with dark hairs except near apex bare. Hair tuft on stem vein yellowish-white. Basal portion of radius fully haired; R1 with dark spinules and hairs; R2 with hairs only. Basal cell absent. Haltere. White with basal portion dark brown. Abdomen. Basal scale yellowish though somewhat darkened along posterior margin, with fringe of yellowish-white hairs. Dorsal surface of abdomen dark brown to brownish-black except segment 2 yellow though tergal plate and narrow area along posterior margin somewhat darkened, moderately covered with dark short to long hairs; tergites of segments 6–9 shiny when illuminated at certain angle of light. Ventral surfaces of segments 2–4 yellow, and those of other segments medium brown to brownish-black; sternal plate on segment 7 undeveloped. Genitalia. Sternite 8 (Fig. 4E) bare medially, with 17–19 medium-long to very long hairs together with few slender short hairs on each side. Ovipositor valves (Fig. 4E) triangular, though rounded medioposteriorly, thin, membranous, moderately covered with microsetae interspersed with 4 short hairs; inner margins straight or very slightly concave, somewhat sclerotized, and moderately separated from each other. Genital fork (Fig. 4F) of usual inverted-Y form, with slender stem; arms wide basally, about twice as wide as apical narrow portion, and moderately folded medially. Paraproct in ventral view (Fig. 4G) shallowly concave anterolaterally, with 5 or 6 sensilla on anteromedial surface; paraproct in lateral view (Fig. 4H) somewhat produced ventrally, 0.63 times as long as wide, with about 20 medium-long to long hairs on ventral and lateral surfaces. Cercus in lateral view (Fig. 4H) short, rounded posteriorly, 0.41 times as long as wide. Spermatheca (Fig. 4I) ellipsoidal, 1.62 times as long as its greatest width, well sclerotized except duct and small area near juncture with duct unsclerotized, and with many fissures on surface; internal setae absent; both accessory ducts slender, subequal in diameter to major one.

**Figure 5.**
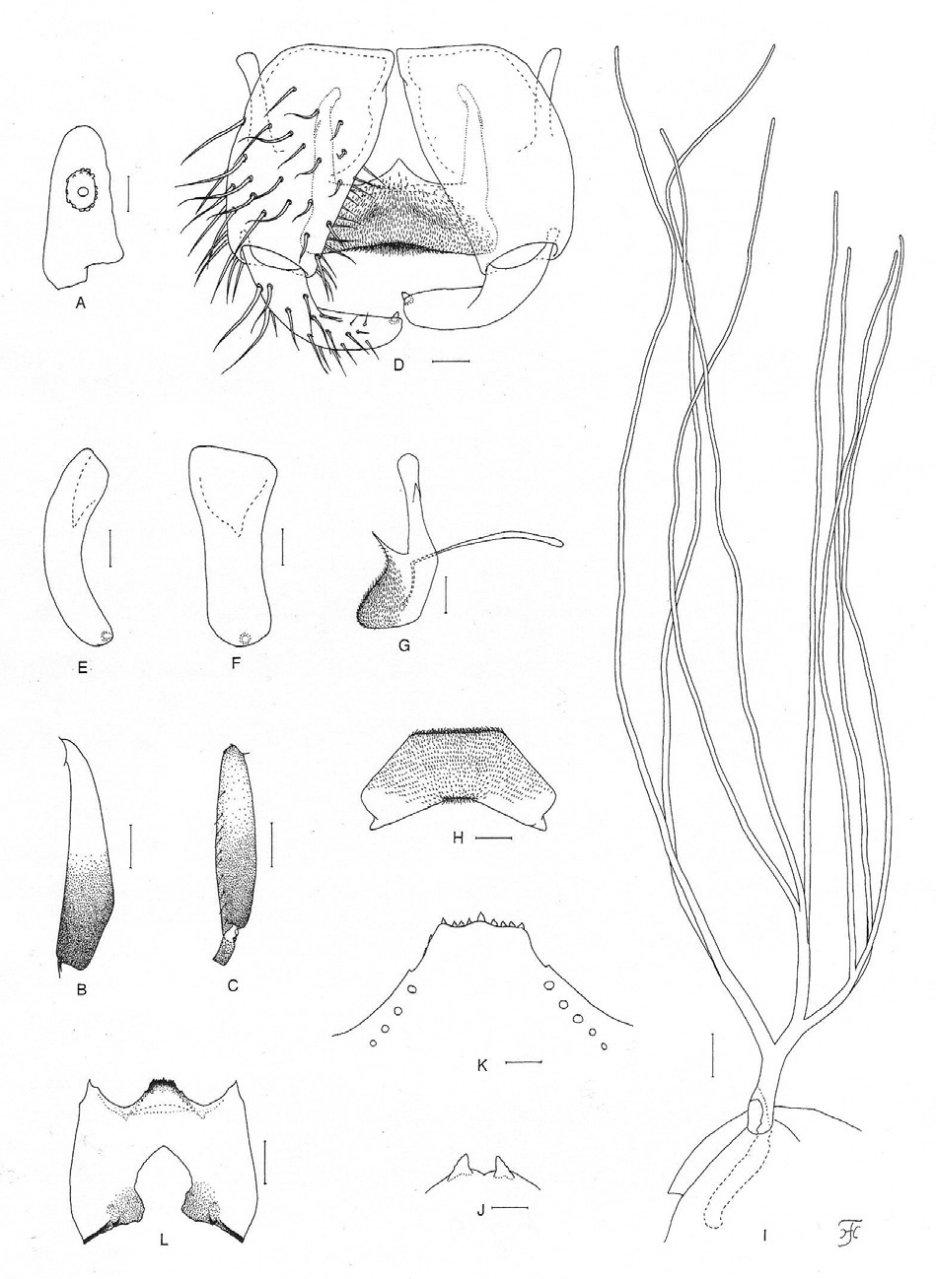
Male, pupa and larva of *Simulium (Gomphostilbia) lurauense* sp. n. **A–H** male **I, J** pupa **K, L** larva **A** 3rd segment of right maxillary palp showing sensory vesicle (front view) **B** left hind tibia (outer view) **C **basitarsus and 2nd tarsomere of left hind leg showing calcipala and pedisulcus (outer view) **D **coxites, styles, ventral plate and median sclerite (ventral view) **E, F** right styles (**E** medial view **F** ventrolateral view) **G** ventral plate and median sclerite (lateral view) **H** ventral plate (end view) **I** left gill filaments (outer view) **J** terminal hooks (end view) **K** hypostoma showing an abnormally small left corner tooth (ventral view) **L** head capsule showing postgenal cleft and hypostoma which is detached posteriorly from head capsule (ventral view). Scale bars. 0.1 mm for B, C, L and I; 0.02 mm for A–H, J and K.

**Male**. Body length 2.5 mm. Head. Somewhat wider than thorax. Upper eye medium brown, consisting of 14 or 15 vertical columns and 14 or 15 horizontal rows of large facets. Face dark brown, grayish-white pruinose. Clypeus brownish-black, whitish pruinose, densely covered with golden yellow scale-like medium-long hairs (mostly directed upward) interspersed with several dark brown simple longer hairs. Antenna composed of scape, pedicel and 9 flagellomeres, dark brown except scape, pedicel and little less than basal 1/2 of 1st flagellomere yellow; 1st flagellomere elongate, 1.73 times as long as 2nd one. Maxillary palp light to medium brown, with 5 segments, proportional lengths of 3rd, 4th, and 5th segments 1.00:1.17:2.43; 3rd segment (Fig. 5A) somewhat widened apically; sensory vesicle (Fig. 5A) ellipsoidal, medium-sized, 0.25–0.29 times as long as 3rd segment, and with small opening. Thorax. Scutum dark brown to black, shiny and thinly grayish-white pruinose on each shoulder, on broad area along each lateral margin and on prescutellar area when illuminated at certain angle of light; scutum densely covered with golden-yellow recumbent short hairs. Scutellum dark brown, with golden-yellow short hairs and dark brown long upright hairs along posterior margin. Postnotum dark brown and bare. Pleural membrane bare. Katepisternum dark brown, moderately covered with fine hairs. Legs. Foreleg: coxa yellow; trochanter light brown except base yellow; femur light brown with apical cap medium brown; tibia light brown except median large portion on outer surface whitish and apical 1/4 dark brown, and with white sheen on outer surface of basal 3/4; tarsus brownish-black; basitarsus moderately dilated, 6.55 times as long as its greatest width. Midleg: coxa medium brown except posterior surface brownish-black; trochanter light brown except basal 1/2 yellow; femur light brown except base yellow and apical cap medium brown (though extreme tip yellow); tibia dark brown except little more than basal 1/3 yellow; tarsus dark brown except base of basitarsus dark yellow. Hind leg: coxa medium brown; trochanter yellow; femur medium brown with base yellow and apical cap dark brown (though extreme tip yellow); tibia (Fig. 5B) dark brown to brownish-black except basal 1/2 yellow; tarsus (Fig. 5C) medium to dark brown except basal 1/2 of basitarsus grayish-white and little less than basal 1/2 of 2nd tarsomere yellowish; basitarsus (Fig. 5C) somewhat enlarged, spindle-shaped, 4.36–4.57 times as long as wide, and 0.81 and 0.71–0.78 times as wide as greatest width of tibia and femur, respectively; calcipala (Fig. 5C) nearly as long as basal width, and 0.36 times as wide as greatest width of basitarsus. Pedisulcus (Fig. 5C) well defined. Wing. Length 1.5 mm. Costa with dark brown spinules as well as dark brown hairs except basal portion with patch of yellowish hairs. Subcosta bare (though 1 hair present on left subcosta of 1 male). Hair tuft on stem vein yellow. Basal portion of radius fully haired; R1 with dark spinules and hairs; R2 with hairs only. Basal cell absent. Haltere. Grayish-white except basal stem darkened. Abdomen. Basal scale dark brown, with fringe of light to medium brown hairs. Dorsal surface of abdomen medium brown to brownish-black except anterior and dorsolateral areas of segment 2 light brown, and moderately covered with dark brown short to long hairs; segments 2 and 5–8 with pair of shiny dorsolateral or lateral patches, of which those on segment 5 confined on posterior 1/2 and less distinct than those on segments 6 and 7. Genitalia. Coxite in ventral view (Fig. 5D) nearly rectangular, 1.81 times as long as its greatest width. Style in ventral view (Fig. 5D) bent inward, slightly tapered from base toward middle, then nearly parallel-sided, rounded apically and with apical spine; style in medial view (Fig. 5E) shorter than coxite (0.80 times as long as coxite), gently bent inward, nearly parallel-sided, with apical spine; style in ventrolateral view (Fig. 5F) moderately tapered from base toward basal 2/5, then nearly parallel-sided and with round apex. Ventral plate in ventral view (Fig. 5D) with body transverse, 0.42 times as long as wide, widened posteriorly, with anterior margin produced anteromedially, and posterior margin slightly concave medially, densely covered with microsetae on ventral surface; basal arms of moderate length, directed forward, then slightly convergent apically; ventral plate in lateral view (Fig. 5G) slightly produced ventrally; ventral plate in end view (Fig. 5H) trapezoidal, though dorsal margin widely concave. Median sclerite (Fig. 5D,G) thin, plate-like, wide. Paramere of moderate size, with 3 distinct long and stout hooks and several smaller ones. Aedeagal membrane moderately setose, slightly sclerotized at base but dorsal plate not well defined. Ventral surface of abdominal segment 10 without distinct hairs near posterior margin. Cercus with 13–17 hairs.

**Pupa**. Body length 2.6 mm. Nearly as in *Simulium (Gomphostilbia) roslihashimi* sp. n. except following characteristics. Thorax. Gill (Fig. 5I) composed of 8 slender thread-like filaments, arranged as [(1+2) (or 2+1)+(1+2)]+2 filaments from dorsal to ventral; common basal stalk medium-long (0.74–0.92 times as long as interspiracular trunk); dorsal and middle triplets share short stalk; dorsal triplet composed of 1 individual and 2 paired filaments with short to medium-long primary stalk and short secondary stalk, and middle triplet composed of 1 individual and 2 paired filaments and bearing medium-long primary stalk and short secondary stalk; ventral paired filaments with medium-long to long stalk which is 1.02–1.36 times as long as common basal stalk and 0.89–1.04 times as long as interspiracular trunk; stalk of ventral pair 1.00–1.08 and 1.27–1.43 times as thick as primary stalks of middle and dorsal triplets, respectively, but 0.88–0.96 times as thick as common stalk of middle and dorsal triplets; primary stalk of dorsal triplet lying against stalk of lower pair at angle of 60–80 degrees when viewed laterally; 3 filaments of dorsal triplet subequal in length (about 2.0 mm long including their own stalks and common basal stalk) and thickness to one another; 3 filaments of middle triplet subequal in length (2.1–2.3 mm long including their own stalks and common basal stalk) and thickness to one another; 2 filaments of ventral pair subequal in length (2.5–2.6 mm long including their own stalk and common basal stalk) and thickness to each other, and 1.38–1.43 and 1.39–1.43 times as thick as those of middle and dorsal triplets, respectively, when compared basally. Abdomen. Dorsally, segments 6–9 each with spine-combs (though those on segment 9 somewhat smaller than those on segment 8) in transverse row and comb-like groups of minute spines on each side; segment 9 with pair of small triangular flat terminal hooks of which outer margin is 1.15 times as long as inner margin and weakly undulated (Fig. 5J). Cocoon. Wall pocket-shaped, thinly and rather roughly woven, somewhat extended ventrolaterally; anterior margin not thickly woven, with dorsal portion not or slightly produced anteriorly when viewed dorsally; 2.5–2.9 mm long by 1.7–1.8 mm wide.

**Mature larva.** Body length 3.9 mm. Body creamy with color markings as follows: thoracic segment 1 encircled with ochreous broad transverse band (though disconnected ventrally), proleg grayish, thoracic segments 2 and 3 light ochreous dorsally and each with distinct ochreous wide areas ventrally, abdominal segments 1–4 each encircled with grayish broad band, abdominal segment 3 also faintly encircled with reddish-purplish band, abdominal segments 5–8 almost entirely covered by sheet of reddish-purplish pigment on dorsal and dorsolateral surfaces (though anterior portion of abdominal segment 5 narrowly unpigmented), from which colored narrow band extends to various extent either laterally or ventrolaterally or even ventrally on each of segments 5–8 (color of band on segments 5, 6 and 8 reddish-purplish and that on segment 7 grayish), and abdominal segment 7 with reddish-purplish transverse broad band ventrally. Cephalic apotome dark yellow, moderately covered with minute setae; head spots very faintly positive. Lateral surface of head capsule yellow except eye-spot region whitish, sparsely covered with minute setae; spots indistinct. Ventral surface of head capsule yellow except darkened area near posterior margin on each side of postgenal cleft, and sparsely covered with minute setae. Antenna composed of 3 segments and apical sensillum, somewhat longer than stem of labral fan; proportional lengths of 1st, 2nd, and 3rd segments 1.00:0.72:0.92. Labral fan with 30 main rays. Mandible with 3 comb-teeth decreasing in length from 1st to 3rd; mandibular serration composed of 2 teeth (1 medium-sized and 1 small); major tooth at acute angle against mandible on apical side; supernumerary serrations absent. Hypostoma (Fig. 5K) with row of 9 apical teeth; median tooth prominent, right corner tooth nearly as long as inner one of intermediate teeth and slightly longer than remaining outer and median teeth, but left corner tooth shorter than outer and median ones of intermediate teeth (this unexpected short corner tooth as well as lack of 2 lateral teeth on left side suggesting anterolateral portion of hypostoma on left side abnormally formed); lateral margin smooth; 4 or 5 hypostomal bristles per side lying slightly divergent posteriorly from lateral margin. Postgenal cleft (Fig. 5L) lanceolate, (its length ratio against postgenal bridge is not accurately attainable due to posterior part of hypostoma which is widely detached from head capsule and folded inward, making it difficult to measure length of postgenal bridge). Cervical sclerite composed of 2 yellow small pieces, not fused to occiput, widely separated medially from each other. Thoracic cuticle bare. Abdominal cuticle almost bare except few posterior segments sparsely to moderately covered with simple minute setae dorsally and dorsolaterally and last segment densely covered with colorless simple setae on each side of anal sclerite. Rectal scales absent. Rectal papilla compound (number of secondary lobules not countable because rectal papilla is withdrawn). Anal sclerite of usual X-form, with anterior arms little longer than posterior ones, broadly sclerotized at base; accessory sclerite absent. Last abdominal segment expanded ventrolaterally forming double bulges on each side, visible as large conical ventral papilla when viewed from side. Posterior circlet with 76 rows of up to 13 hooklets per row.

**Type specimens**. Holotype male (with associated pupal exuviae and cocoon) (preserved in 80% ethanol) reared from pupa, collected from a river (Sungai Lurau) (width 10–12 m, water temperature 22.0°C, partially shaded, altitude 530 m, 03°18'22.9"N, 101°52'50.0"E) moderately to rapidly flowing, Janda Baik, Pahang, Malaysia, 22. II. 2011, by M. Sofian-Azirun and H. Takaoka. Paratype: 1 female (with associated pupal exuviae and cocoon) (preserved in 80% ethanol), collected from a small stream (width 0.3–0.5 cm, water temperature 23.0°C, exposed to sun, altitude 582 m, 05°33'95.8"N, 101°36'65.2"E), very slowly flowing in a flat bushy area before joining to Selaur River, Temengor, Perak, 26. IV. 2011, by Z. Ya’cob; 1 male (with associated pupal exuviae and cocoon) (preserved in 80% ethanol) and 1 mature larva (preserved in acetic alcohol), collected from a small stream (width 1–3 m, water temperature 22.0°C, shaded, altitude 380 m, 03°43'36.4"N, 101°47'33.9"E), moderately flowing in a natural forest, along the road from Raub to Fraser’s Hill, Pahang, 12. IV. 2011, by M. Sofian-Azirun, Z. Ya’cob and H. Takaoka.

**Biological notes**. In Janda Baik, the pupa of this new species was collected from a tree leaf trailing in the water. Associated species were *Simulium (Simulium) hirtinervis* Edwards, 1928, *Simulium (Simulium) nobile* de Meijere, 1906 and *Simulium (Simulium) tani* Takaoka & Davies, 1995. In the stream between Raub and Fraser’s Hill, the other pupa was collected from a trailing grass, and associated species were *Simulium (Simulium) bishopi* Takaoka & Davies, 1995, *Simulium (Gomphostilbia) decuplum* Takaoka & Davies, 1995 and *Simulium (Simulium) tani*. In Perak, the pupa was collected from a trailing grass and associated species were *Simulium (Gomphostilbia) whartoni* and *Simulium (Simulium) tani*.

#### Etymology.

 The species name *lurauense* refers to the name of the river where this new species was collected for the first time.

#### Remarks.

 *Simulium (Gomphostilbia) lurauense* sp. n. is most similar to *Simulium (Gomphostilbia) sofiani* recently described from Cameron Highland (Takaoka et al. 2011) in sharing the elongate female sensory vesicle (Fig. 4A), the yellowish-white hairs on the base of the costal vein and on the stem vein, the greater number of enlarged male upper-eye facets, the narrow, spindle-shaped male hind basitarsus (Fig. 5C), the trapezoidal male ventral plate when viewed posteriorly (Fig. 5H) and the pupal terminal hook of narrow triangular shape (Fig. 5J). However, this new species is distinguished from *Simulium (Gomphostilbia) sofiani* in the female by the arms of the genital fork which are wide basally and narrowed apically (Fig. 4F), in the male by the medium-sized sensory vesicle (Fig. 5A), in the pupa by the medium-long common basal stalk of the gill (Fig. 5I) and in the larva by the abdominal segments 5–8 with reddish-purplish markings dorsally (cf., the arms of the female genital fork are narrow throughout its length, the male sensory vesicle is globular and small, 0.15 times as long as the maxillary palpal segment 3, the common basal stalk of the pupal gill is long, 1.0–1.2 times as long as the interspiracular trunk, and the dorsal surfaces of larval abdominal segments 5–8 are faintly greenish in *Simulium (Gomphostilbia) sofiani*).

This new species is distinguished from the other species of the *ceylonicum* species-group reported from Peninsular Malaysia as shown in the keys.

Among the known species of the *ceylonicum* species-group reported from other Asian countries, *Simulium (Gomphostilbia) dudgeoni* Takaoka & Davies, 1995, from Hong Kong is similar to *Simulium (Gomphostilbia) lurauense* sp. n. in having the nearly similar number of enlarged male upper-eye facets, the spindle-shaped male hind basitarsus, and the ventral plate which is widened posteriorly when viewed ventrally, but is distinguished by the almost dark male hind tibia (Takaokaet al. 1995). In addition, *Simulium (Gomphostilbia) namense* Takaoka, 1989, from Myanmar shows a similar number of enlarged male upper-eye facets but differs by having the dark brown male scutum with three longitudinal vittae, the wedge-shaped male hind basitarsus and the ventral plate slightly narrowed posteriorly when viewed ventrally (Takaoka 1989).

## Keys to three species-groups of the subgenus Simulium (Gomphostilbia) reported from Peninsular Malaysia

The three species-groups are morphologically indistinguishable from one another in the pupal and larval stages.

### Females

**Table:** 

1	Antenna with 7 or 8 flagellomeres	*varicorne* species-group
–	Antenna with 9 flagellomeres	2
2	Hind tibia whitish on basal 1/3 to 3/4 and without subbasal dark spot	*ceylonicum* species-group
–	Hind tibia mostly brownish with base whitish, or hind tibia whitish on basal 1/2 or more and with subbasal dark spot	*batoense* species-group

### Males

**Table:** 

1	Antenna with 7 or 8 flagellomeres	*varicorne* species-group
–	Antenna with 9 flagellomeres	2
2	Hind basitarsus enlarged, wedge- or spindle-shaped	*ceylonicum* species-group
–	Hind basitarsus slender, parallel-sided	*batoense* species-group

## Keys to species of the ceylonicum species-group of the subgenus Simulium (Gomphostilbia) reported from Peninsular Malaysia

### Females 

(*Simulium hoiseni* is not included because its female remains unknown)

**Table:** 

1	Hairs on stem vein black	*Simulium sheilae*
–	Hairs on stem vein whitish-yellow or yellow	2
2	Sensory vesicle elongate, 0.50–0.62 times as long as maxillary palpal segment 3	3
–	Sensory vesicle medium-long, 0.22–0.30 times as long as maxillary palpal segment 3	4
3	Arms of genital fork narrow throughout its length	*Simulium sofiani*
–	Arms of genital fork with wide basal portion, about twice as wide as apical narrow portion	*Simulium lurauense* sp. n.
4	Outer margin of mandible without teeth	*Simulium roslihashimi* sp. n.
–	Outer margin of mandible with teeth	5
5	Hind tibia whitish-yellow on basal 1/2	*Simulium longitruncum*
–	Hind tibia whitish-yellow on basal 2/3	*Simulium asakoae*

### Males

**Table:** 

1	Hairs on stem vein black	*Simulium sheilae*
–	Hairs on stem vein yellow	2
2	Hind basitarsus wedge-shaped, and as wide as or little wider than hind tibia	3
–	Hind basitarsus spindle-shaped, and narrower than hind tibia	4
3	Antenna yellow to ochreous, though apical 4 or 5 flagellomeres darkened	*Simulium hoiseni*
–	Antenna brown except scape, pedicel and base of 1st flagellomere yellow	*Simulium asakoae*
4	Antenna almost entirely yellow	*Simulium roslihashimi*
–	Antenna brown except scape, pedicel and base of 1st flagellomere yellow	5
5	Hind basitarsus 0.9 times as wide as hind tibia; body of ventral plate slightly narrowed posteriorly when viewed ventrally	*Simulium longitruncum*
–	Hind basitarsus 0.8 times as wide as hind tibia; body of ventral plate somewhat widened posteriorly when viewed ventrally	6
6	Sensory vesicle short, 0.15 times as long as maxillary palpal segment 3	*Simulium sofiani*
–	Sensory vesicle medium-long, 0.25–0.29 times as long as maxillary palpal segment 3	*Simulium lurauense*

### Pupae

**Table:** 

1	Gill with 6 filaments	*Simulium hoiseni*
–	Gill with 8 filaments	2
2	Common basal stalk of gill as long as or longer than interspiracular trunk	3
–	Common basal stalk of gill shorter than interspiracular trunk	4
3	Dorsal and middle triplet groups of gill filaments sharing common stalk	*Simulium sofiani*
–	Dorsal triplet group of gill filaments arising directly from common basal stalk	*Simulium longitruncum*
4	Abdominal segments 1 and 2 dark grayish, with minute tubercles	*Simulium asakoae*
–	Abdominal segments 1 and 2 transparent or light yellow, without tubercles	5
5	Dorsal and middle triplets of gill filaments sharing very short common stalk; common basal stalk shorter than 3/5 of interspiracular trunk	*Simulium sheilae*
–	Dorsal and middle triplets of gill filaments sharing short to medium-long common stalk; common basal stalk longer than 3/5 of interspiracular trunk	6
6	Terminal hooks narrow, with outer margin slightly longer than inner margin and undulate	*Simulium lurauense*
–	Terminal hooks wide, with outer margin over twice as long as inner margin and crenulate	*Simulium roslihashimi*

### Larvae 

(*Simulium hoiseni* is not included because its larva remains unknown)

**Table:** 

1	Postgenal cleft very long, with its apex almost reaching posterior border of hypostoma	*Simulium sheilae*
–	Postgenal cleft medium-long, leaving space (i.e., postgenal bridge) between itsapex and posterior border of hypostoma	2
2	Ventral surface of head capsule darkened around postgenal cleft	*Simulium asakoae*
–	Ventral surface of head capsule not darkened around postgenal cleft	3
3	Abdominal segments 5–8 with reddish-brown or reddish-purplish markings on dorsal surface	4
–	Abdominal segments 5–8 without such reddish markings on dorsal surface	5
4	Dorsal surfaces of abdominal segments 5 and 6 almost entirely reddish-purplish	*Simulium lurauense*
–	Dorsal surfaces of abdominal segments 5 and 6 each with reddish-brown W-shaped transverse band	*Simulium roslihashimi*
5	Body length 4.2–4.6 mm; posterior circlet with 80 rows of up to 12 hooklets per row	*Simulium sofiani*
–	Body length 5.0–5.4 mm; posterior circlet with 86–96 rows of up to 15 hooklets per row	*Simulium longitruncum*

## Supplementary Material

XML Treatment for Simulium (Gomphostilbia) roslihashimi

XML Treatment for Simulium (Gomphostilbia) lurauense
